# Supplementary material for: All-at-once RNA folding with 3D motif prediction framed by evolutionary information
Source: Nat Methods. 2025 Oct 3;22(10):2094–106. doi: 10.1038/s41592-025-02833-w (PMC12510878; doi:10.1038/s41592-025-02833-w)
Supplement: Supplementary file 3 — Source code to run CaCoFold-R3D. Data and results. [file 41592_2025_2833_MOESM3_ESM.gz › supplemental_material/Figures/Figure_S1.pdf]

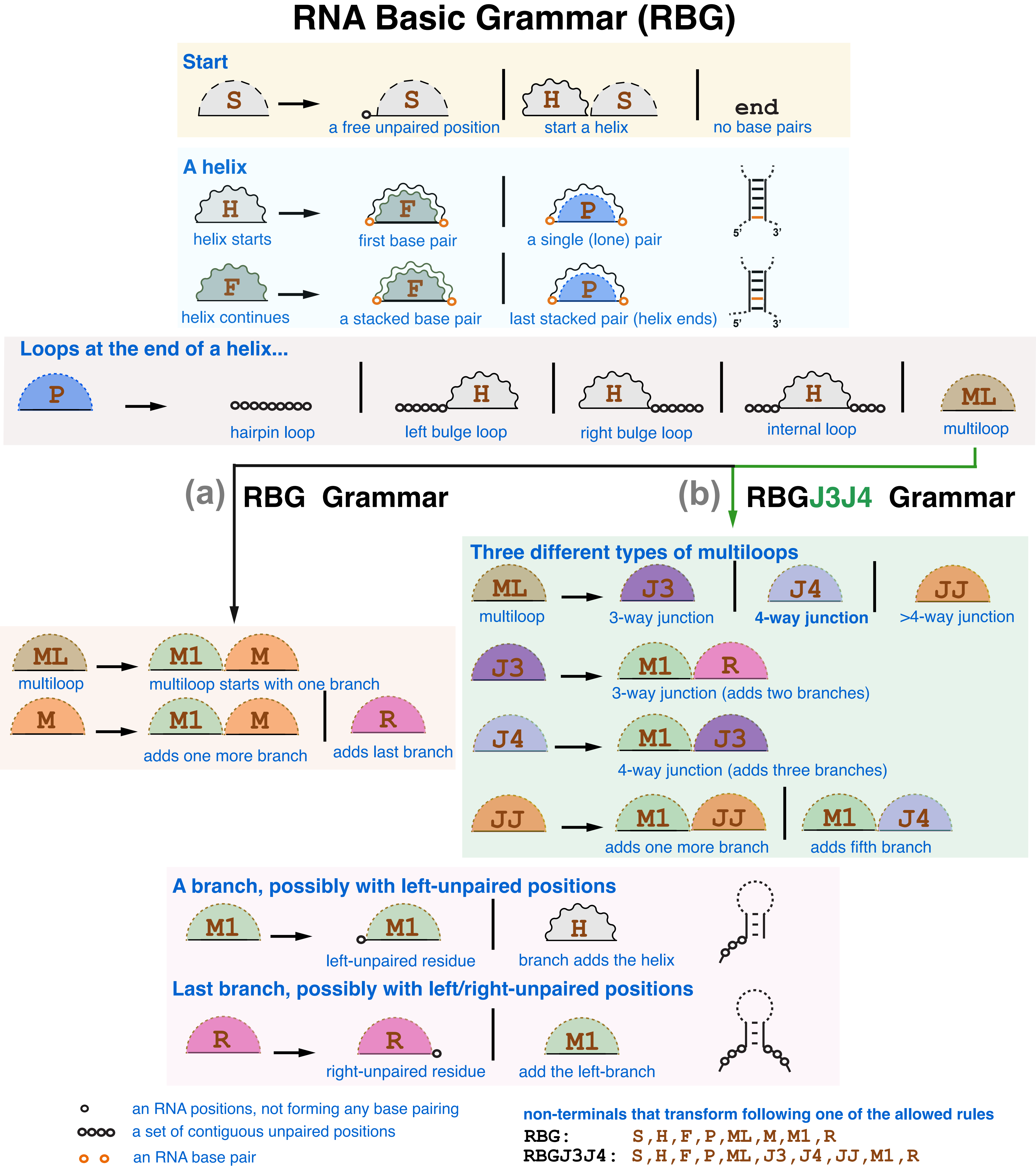

RNA Basic Grammar (RBG)
Start
S → S a free unpaired position | H S start a helix | end no base pairs
A helix
H helix starts → F first base pair | P a single (lone) pair
5' 3'
F helix continues → F a stacked base pair | P last stacked pair (helix ends)
5' 3'
Loops at the end of a helix...
P → hairpin loop | H left bulge loop | H right bulge loop | H internal loop | ML multiloop
(a) RBG Grammar
(b) RBGJ3J4 Grammar
ML multiloop → M1 M multiloop starts with one branch
M → M1 M adds one more branch | R adds last branch
Three different types of multiloops
ML multiloop → J3 3-way junction | J4 4-way junction | JJ >4-way junction
J3 → M1 R 3-way junction (adds two branches)
J4 → M1 J3 4-way junction (adds three branches)
JJ → M1 JJ adds one more branch | M1 J4 adds fifth branch
A branch, possibly with left-unpaired positions
M1 → M1 left-unpaired residue | H branch adds the helix
Last branch, possibly with left/right-unpaired positions
R → R right-unpaired residue | M1 add the left-branch
an RNA positions, not forming any base pairing
a set of contiguous unpaired positions
an RNA base pair
non-terminals that transform following one of the allowed rules
RBG: S,H,F,P,ML,M,M1,R
RBGJ3J4: S,H,F,P,ML,J3,J4,JJ,M1,R
